# PBMCs transcriptome profiles identified breed-specific transcriptome signatures for PRRSV vaccination in German Landrace and Pietrain pigs

**DOI:** 10.1371/journal.pone.0222513

**Published:** 2019-09-19

**Authors:** Md. Aminul Islam, Christiane Neuhoff, Sharmin Aqter Rony, Christine Große-Brinkhaus, Muhammad Jasim Uddin, Michael Hölker, Dawit Tesfaye, Ernst Tholen, Karl Schellander, Maren Julia Pröll-Cornelissen

**Affiliations:** 1 Department of Animal Breeding and Husbandry, Institute of Animal Science, University of Bonn, Endenicher Allee 15, Bonn, Germany; 2 Department of Medicine, Faculty of Veterinary Science, Bangladesh Agricultural University, Mymensingh, Bangladesh; 3 Department of Parasitology, Faculty of Veterinary Science, Bangladesh Agricultural University, Mymensingh, Bangladesh; 4 School of Veterinary Science, The University of Queensland, Gatton campus, Brisbane, QLD, Australia; 5 Teaching and Research Station on Frankenforst, Faculty of Agriculture, University of Bonn, Königswinter, Germany; University of Illinois, UNITED STATES

## Abstract

Porcine reproductive and respiratory syndrome (PRRS) is a devastating viral disease affecting the swine industry worldwide. Genetic variation in host immunity has been considered as one of the potential determinants to improve the immunocompetence, thereby resistance to PRRS. Therefore, the present study aimed to investigate the breed difference in innate immune response to PRRSV vaccination between German Landrace (DL) and Pietrain (Pi) pigs. We analyzed microarray-based transcriptome profiles of peripheral blood mononuclear cells (PBMCs) collected before (0 h) and 24 h after PRRSV vaccination from purebred DL and Pi pigs with three biological replicates. In total 4,269 transcripts were identified to be differentially expressed in PBMCs in at least any of four tested contrast pairs (i.e. DL-24h vs. DL-0h, Pi-24h vs. Pi-0h, DL-0h vs. Pi-0h and DL-24h vs. Pi-24h). The number of vaccine-induced differentially expressed genes (DEGs) was much higher (2,459) in DL pigs than that of Pi pigs (291). After 24 h of PRRSV vaccination, 1,046 genes were differentially expressed in PMBCs of DL pigs compared to that of Pi (DL-24h vs. Pi-24h), indicating the breed differences in vaccine responsiveness. The top biological pathways significantly affected by DEGs of both breeds were linked to immune response functions. The network enrichment analysis identified ADAM17, STAT1, MMS19, RPA2, BAD, UCHL5 and APC as potential regulatory genes for the functional network of PRRSV vaccine response specific for DL; while FOXO3, IRF2, ADRBK1, FHL3, PPP2CB and NCOA6 were found to be the most potential hubs of Pi specific transcriptome network. In conclusion, our data provided insights of breed-specific host transcriptome responses to PRRSV vaccination which might contribute in better understanding of PPRS resistance in pigs.

## Introduction

Porcine reproductive and respiratory syndrome (PRRS) is one of the most economically important viral diseases of swine industry worldwide. PRRS is caused by a positive sense, single-stranded RNA virus PRRS virus (PRRSV) having two genetically diverse strains namely Type 1 (European) and Type 2 (North American) [1]. The clinical outcome of PRRSV infection varies widely from a mild, asymptomatic illness to a severe, clinical disease, depending on the virulence of the virus and the immune status of the host [2]. PRRSV of either genotype seems to inherently develop an imbalanced immune response characterized by aberrant interferon (IFN) responses [3]. Variability of host immunity is likely responsible for inconsistency of the clinical outcomes seen upon PRRSV challenge either to naive or previously immunized pigs [4]. Therefore, severity of PRRSV infection is determined by hosts’ ability to overcome the inherent propensity of PRRSV in preventing timely onset of innate immune response.

Innate immunity is the front-line host defense mechanism, which is typically developed within hours of antigen exposure and may persist up to a few days [5]. An adequate and timely activation of the innate immune system is essential for mounting a durable, protective immunity [6]. Genes regulating the innate immune response to pathogenic infection are likely strong candidates for host resistance to disease [5]. Since the vaccine antigen mimics a natural infection in term of activating host defense, innate immunity to vaccination has been considered as a potential indirect measure of host resistance [7]. Innate immunity related genes, in particular, members of the guanylate-binding protein (GBP) gene family have been found as potential candidate for host resistance to PRRSV [8, 9]. A major quantitative trait locus (QTL) for PRRS resistance has been identified on Sus scrofa chromosome 4 (SSC4) where genes of the GBP family are located [9]. Moreover, single nucleotide polymorphisms (SNPs) within GBP5 [10], GBP1 [11] and ubiquitin specific protease 18 (USP18) [12] gene have been reported to be associated with host resistance to PRRSV infection. Therefore, identification of genes and their expression regulation associated with innate immunity to PRRSV vaccination are crucial for the improvement of host genetic resistance.

Breed is one of the potential host determinants affecting immune responses to a variety of pathogens or stressors in pigs. The existence of breed differences in relative resistance to PRRSV infection in pigs has been reported in several studies [13–18]. Variations in host innate immunity to European type PRRSV infection have been explored between Landrace and Pietrain (Pi) pigs through global gene expression profiling of in vitro PRRSV infected pulmonary alveolar macrophages (PAMS) [15]. Christopher-Hennings et al. [16] compared the presence of virus in serum, semen and peripheral blood mononuclear cells (PBMCs) over time in adult Hampshire (n = 3), Yorkshire (n = 3), and Landrace (n = 2) boars inoculated with a PRRSV field isolate (SD-23983). Variations observed in the immune responses to PRRSV with a few animals of each breed tested [16] reinforced the possibility of detecting statistically significant breed-differences even with a relatively smaller study population. A population of lean type pigs showed higher susceptibility to PRRSV as compared to that of non-lean type pigs [17], similar results were also reported by a comparative evaluation of PRRSV infection between German miniature and Pi pigs [18]. In a recent study, we also observed remarkable differences between Duroc and Pi pigs in terms of transcriptome profiles of lung dendritic cells after in vitro PRRSV infection [19]. All these above-mentioned works have raised the evidence for genetic variation in host transcriptional response to PRRSV vaccination among porcine breeds.

In order to characterize host responses, several studies evaluated the transcriptome profiles of respiratory tissues/cells following in vitro and in vivo PRRSV infection [12–15, 20], perhaps because of primary site of viral replication. It has been reported that intramuscularly administered PRRSV vaccine antigens bypass the lung tissue and enter into blood circulation and initiate the immune reaction [21]. Therefore, blood-based investigation of molecular mechanisms of host-vaccine interaction is worthwhile. Blood transcriptomics could provide a quick insight into the complex biological processes linking between host genotypes and vaccine responses [22, 23]. Furthermore, breed variation in PRRSV vaccine-induced transcriptome modifications at blood level among the porcine breeds has not yet been entirely explored. Our previous study revealed temporal patterns of transcriptome alterations over the first three days of immunization with a peak response at 24 h post vaccination in pigs [24]. Thus, the time point 24 h post vaccination was selected for identifying transcriptome signatures of innate immune response to PRRSV vaccine. At 24 h post PRRSV vaccination, host genetic variation in PRRSV between DL pigs [24] and Pi pigs [25] were observed. To investigate whether host genetic variation impacts on vaccine-induced innate immunity, we compared the global gene expression profiles of PBMCs collected immediately before and at 24 h post PRRSV vaccination in purebred DL and Pi pigs.

## Material and methods

### Ethics statement

The research proposal was approved by the Veterinary and Food Inspection Office, Siegburg, Germany (ref. 39600305-547/15).

### Study design and data description

To explore the breed differences in PRRSV vaccine-induced gene expression profiles between DL and Pi pigs, we analyzed 12 microarray data retrieved from two of our previous studies [24, 25]. The microarray data analyzed here were generated in PBMCs collected before and at 24 h post PRRSV vaccination in three individual DL and Pi piglets. The raw data for DL and Pi pigs are available through NCBI-GEO accession number GSE76254 [24] and GSE84516 [25], respectively. Study piglets were female litter mates from both purebred DL and Pi breed, and were housed at the Teaching and Research Station at Frankenforst, University of Bonn, Germany. Piglets were maintained under same husbandry condition and were immunized with the live attenuated PRRSV vaccine of EU strain (Porcillis PRRS, MSD Animal Health, Germany) with a primary injection at 28 days old. Piglets were confirmed sero-negative at the time of primary vaccination through PRRSV-specific antibody ELISA (PRRSV-AK screening, SynLab Vet. GmbH, Standort Augsburg, Germany) screening. The whole blood samples collected at 0 and 24 h post vaccination were subjected to PBMCs isolation through density gradient centrifugation with Histopaque1077 (Sigma-Aldrich, Germany). The total RNA was extracted from PBMCs using the miRNeasy mini kit (Qiagen, Hilden, Germany) according to the manufacturers protocol along with on column DNase treatment (Qiagen, Hilden, Germany). After quality control, about 100 ng of total RNA was processed to synthesize the biotin-labeled sense strand cDNA probes using the GeneChip WT PLUS Reagent kit (Affymetrix Inc., Santa Clara, CA, USA) according to the manufacturer’s protocol. The microarray target probes were hybridized onto the GeneChip Porcine Gene 1.0 ST array of 81/4 format (Affymetrix Inc., Santa Clara, CA, USA) followed by staining, washing and scanning using the Affymetrix GeneChip array processing facility at the Life & Brain Centre, University Hospital Bonn, Germany. The microarray expressions of both datasets were technically validated through qRT-PCR expression of selected differentially expressed genes in the same RNA sample as used for microarray hybridization.

### Statistical analysis of microarray data

The raw intensity data was processed for background correction and normalization with R/Bioconductor software (v 3.1.2). The RMA (Robust Multi-array Average) based quantile normalization of microarray data were performed using the oligo package [26]. For the differential expression analysis, normalized microarray dataset was prepared for four pairwise comparisons: DL-24h vs. DL-0h, Pi-24h vs. Pi-0h, DL-0h vs. Pi-0h and DL-24h vs. Pi-24h. Differentially expressed genes were determined using the linear analysis of microarray technique from the limma package [27] with empirical Bayes adjustment to the variance, followed by Benjamini and Hochberg (BH) correction for multiple testing [27, 28]. Threshold criteria for genes to be considered differentially expressed were set as false discovery rate (FDR) of <0.05 and log2 fold-change either >1.5 or <-1.5. The hierarchical clustered heat map was generated using the heatmap.2 function of ggplots package in R.

### Functional annotation of differentially expressed genes

For the biological interpretation of the altered PBMC-transcriptomes between the two breeds, significantly over-represented gene ontology (GO) terms and biological pathways were explored using the InnateDB pathway analysis tool [29]. The InnateDB platform implements a hypergeometric algorithm with Benjamini- Hochberg (BH) multiple test correction method for overrepresentation analysis. First, the differentially expressed genes (DEGs) from microarray data were converted to their human ensembl orthologues using the biological DataBase network (bioDBnet) tool [30]. Then a list of ensembl gene identifiers was uploaded in InnateDB web and the over-representation analysis was performed. GO terms and pathways were considered significantly over-represented with an FDR of <0.05.

### Network analysis for differentially expressed genes

To identify the potential regulatory genes of vaccine mediated immunity in a breed specific manner, we performed network enrichment analysis with the DEGs more abundant in vaccinated PBMCs of DL compared to that of Pi pigs and vice versa using the NetworkAnalyst online tool [31]. Human orthologous gene ensemble of the DEGs were imported as seed genes and a default network was constructed based on the Walktrap algorithm taking only direct interaction of seed genes (first-order interactors). The network size was then adjusted for <500 seeds and nodes between 200 and 2000 using the reduce panel for high-performance visualization. Two topological measures such as degree (number of connections to other nodes) and betweenness centrality (number of shortest paths going through the node) were taken into account for detecting highly potential hubs that could regulate the entire network. The degree and betweenness estimates attributed to the diameter of particular nodes. Therefore, larger diameter of a node indicates higher potential to be the network hub. In addition, weighted network-based module detection was performed to cluster the genes of similar biological functions. The P value of a given network module was calculated using a Wilcoxon rank-sum test of the”internal” (edges within in a module) and”external” (edges connecting the nodes of other modules) degrees.

## Results

In order to get comprehensive insights of vaccine-induced transcriptome differences between piglets of DL and Pi breed, we compared the whole transcriptome profiles of PBMCs collected immediately before (0 h) and 24 h after primary PRRSV vaccination. The transcriptome profiling was performed with three individual biological replications for each sampling time points in both breed groups using Affymetrix GeneChip Porcine Gene 1.0 ST array containing 394,580 probe sets representing a total of 19,212 known genes.

### Abundance of differentially expressed genes in PBMCs after PRRSV vaccination

**Fig 1:**
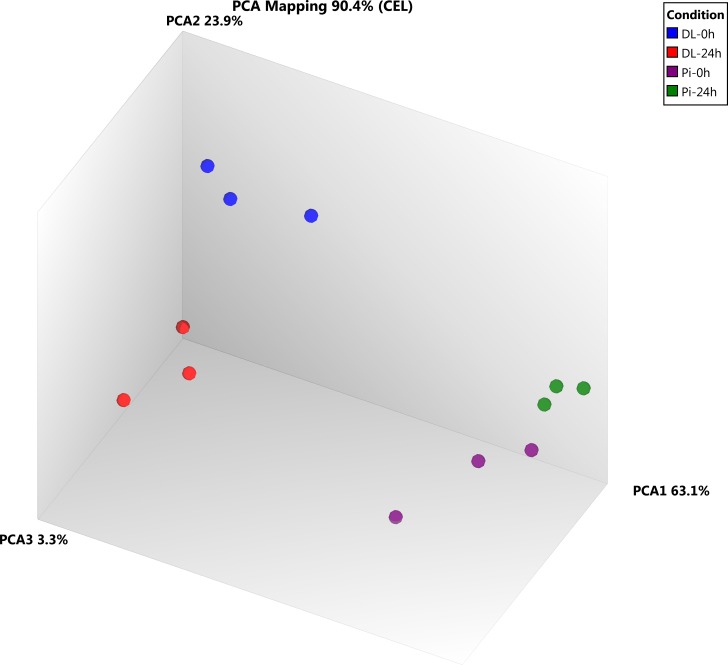
Principal component analysis (PCA) plot showing the sample characteristics based on microarray expression data. Each solid circle indicates the normalized gene expression of one microarray sample and the color of the circle indicate treatment condition.

**Fig 2:**
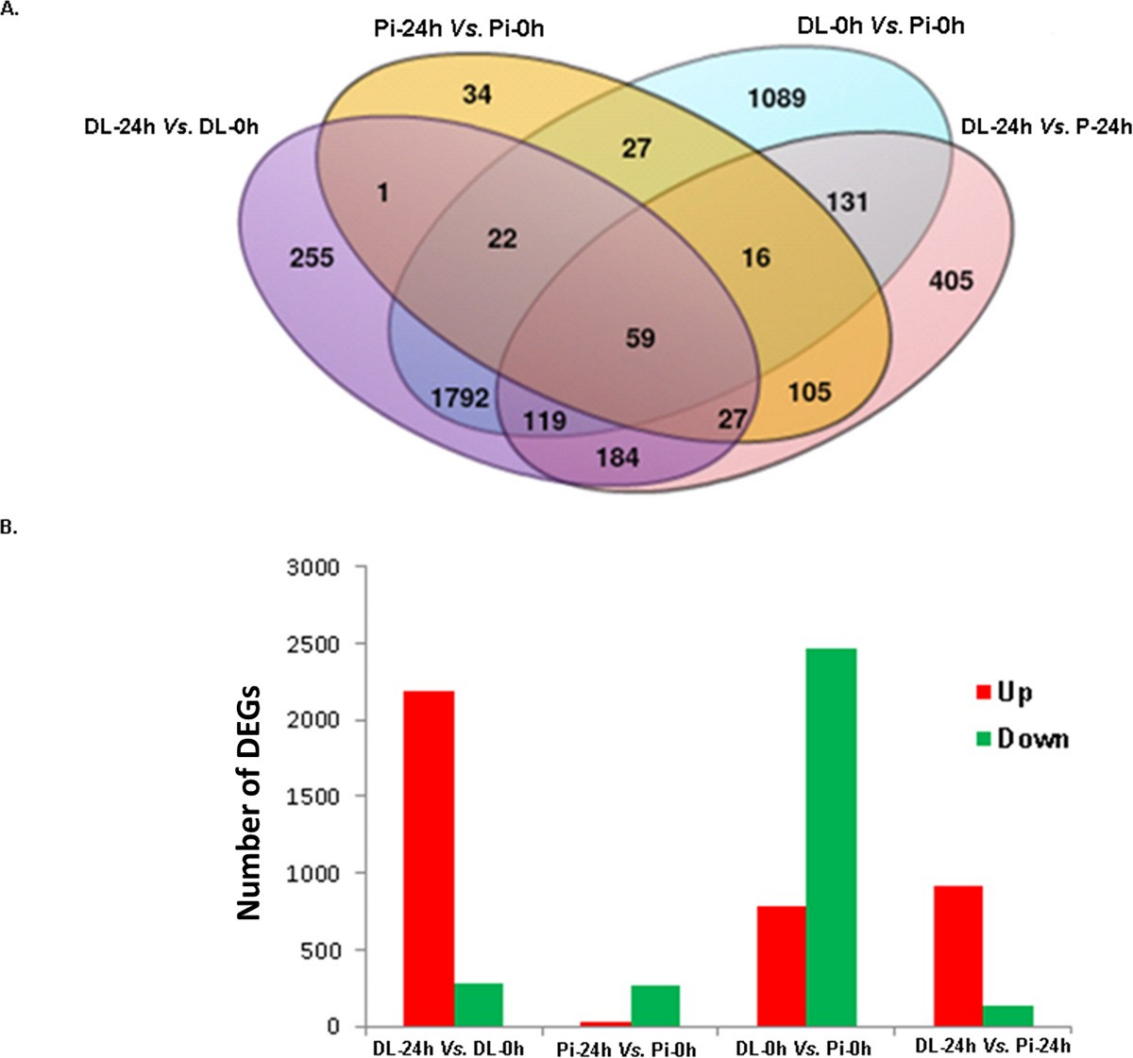
Number of DEGs after PRRSV vaccination. (A) The intersecting venn diagram demonstrates the number of DEGs identified at four contrast pairs such as PBMCs of DL pigs between pre and 24 h post vaccination (DL-24h vs. DL-0h); PBMCs of PI pigs between pre and 24 h post vaccination (Pi-24h vs. Pi-0h); unvaccinated PBMCs between DL and Pi pigs (DL-0h vs. Pi-0h), and vaccinated PBMCs between DL and Pi pigs (DL-24h vs. Pi-24h). (B) The bar graphs depict the proportion of DEGs showed their expression either up regulated (red bars) or down regulated (green bars) direction at four contrast pairs tested.

At first, the characteristics of microarray expression data were determined using the principal component analysis (PCA) plot (Fig 1). Interestingly, the results showed that microarray data were very similar within a sampling time point of each breed, and clustered together indicating the homogeneity of transcriptome profiles of a particular treatment condition (Fig 1). On the other hand, samples from two breeds were located in two clearly separate zones (Fig 1). Then, gene transcripts were considered differentially expressed with thresholds set as FDR <0.05 and log2 fold-change either >1.5 or <-1.5. Four contrast pairs namely PBMCs of DL pigs between before and 24 h post vaccination (DL-24h vs. DL-0h), PBMCs of Pi pigs between before and 24 h post vaccination (Pi-24h vs. Pi-0h), unvaccinated PBMCs between DL and Pi pigs (DL-0h vs. Pi-0h) and vaccinated PBMCs between DL and Pi pigs (DL-24h vs. Pi-24h) were taken into consideration for differential expression analysis. Following statistical analysis of RMA normalized expression data obtained from both breeds together, 4,269 transcripts were found to be differentially expressed in at least one of the four contrast pairs, while 2,459, 291, 3255 and 1,046 DEGs were identified in the pairwise comparison of DL-24h vs. DL-0h, Pi-24h vs. Pi-0h, DL-0h vs. Pi-0h and DL-24h vs. Pi-24h, respectively. Notably, 59 genes were differentially expressed in all four contrast pairs irrespective of vaccine responses and breed differences (Fig 2A). A total of 2,350 genes were differentially expressed in PBMCs at 24 h after vaccination in DL pigs while only 182 genes were differentially expressed in Pi pigs after vaccination. Furthermore, a total of 721 DEGs showed more abundance in PBMCs of vaccinated DL pigs compared to that of Pi pigs, among which 405 genes were altered solely by breed differences (Fig 2A).

### Global expression patterns of DEGs between DL and Pietrain pigs

**Fig 3:**
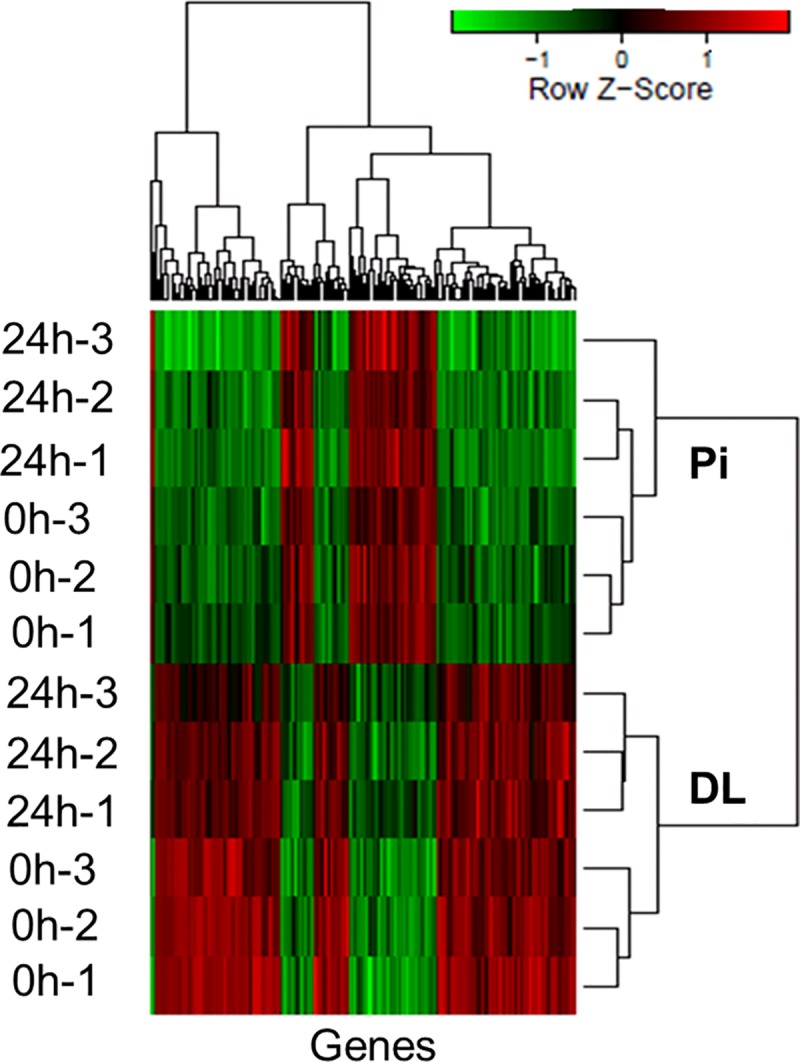
Hierarchical heat map showing differential gene expression over the contrast pairs. The figure includes DEGs between vaccinated PBMCs of DL pigs compared to that of Pi pigs. The normalized log2 transformed values determined by Affymetrix GeneChip porcine gene 1.0 ST array in PBMCs collected at 0 and 24 h post PRRSV vaccination both in DL and Pi pigs. The cutoff values of log2 fold change as either >1.5 or <-1.5 and FDR <0.05 were considered for statistical significance. Each column represents one array from each of replicate piglets.

In PBMCs of vaccinated DL pigs, a large number of DEGs (2,186) were up-regulated compared to the down-regulated ones (273) (Fig 2B). On the other hand, the majority of the altered genes (260) in PBMCs of vaccinated Pi pigs were down-regulated, and only 31 genes were up-regulated. In breed comparison before vaccination, 783 genes were up-regulated in PBMCs of DL compared to Pi and 2,472 genes were down-regulated in DL compared to that of Pi pigs, respectively. In breed comparison after vaccination, relatively higher number (933) of up-regulated genes were observed in vaccinated PBMCs of DL pigs compared to those of Pi pigs (133) (Fig 2B). The range of log fold changes of DEGs in the four contrasts includes -3.87 to 5.12; -4.71 to 3.63; -5.87 to 6.41 and -3.89 to 6.72 in the contrasts of DL-24h vs. DL-0h; Pi-24h vs. Pi-0h; DL-0h vs. Pi-0h and DL-24h vs. Pi-24h, respectively. The hierarchical heatmap (Fig 3) demonstrated the distinct patterns of differential gene expression in PBMCs of DL and Pi pigs. Sample dendrogram revealed that replicates were clustered together within each treatment block tested. The DEGs were clustered in five major groups based on the similarities of biological functions (Fig 3).

### Mutually inclusive transcriptome response to PRRSV vaccine between DL and Pi pigs

**Fig 4:**
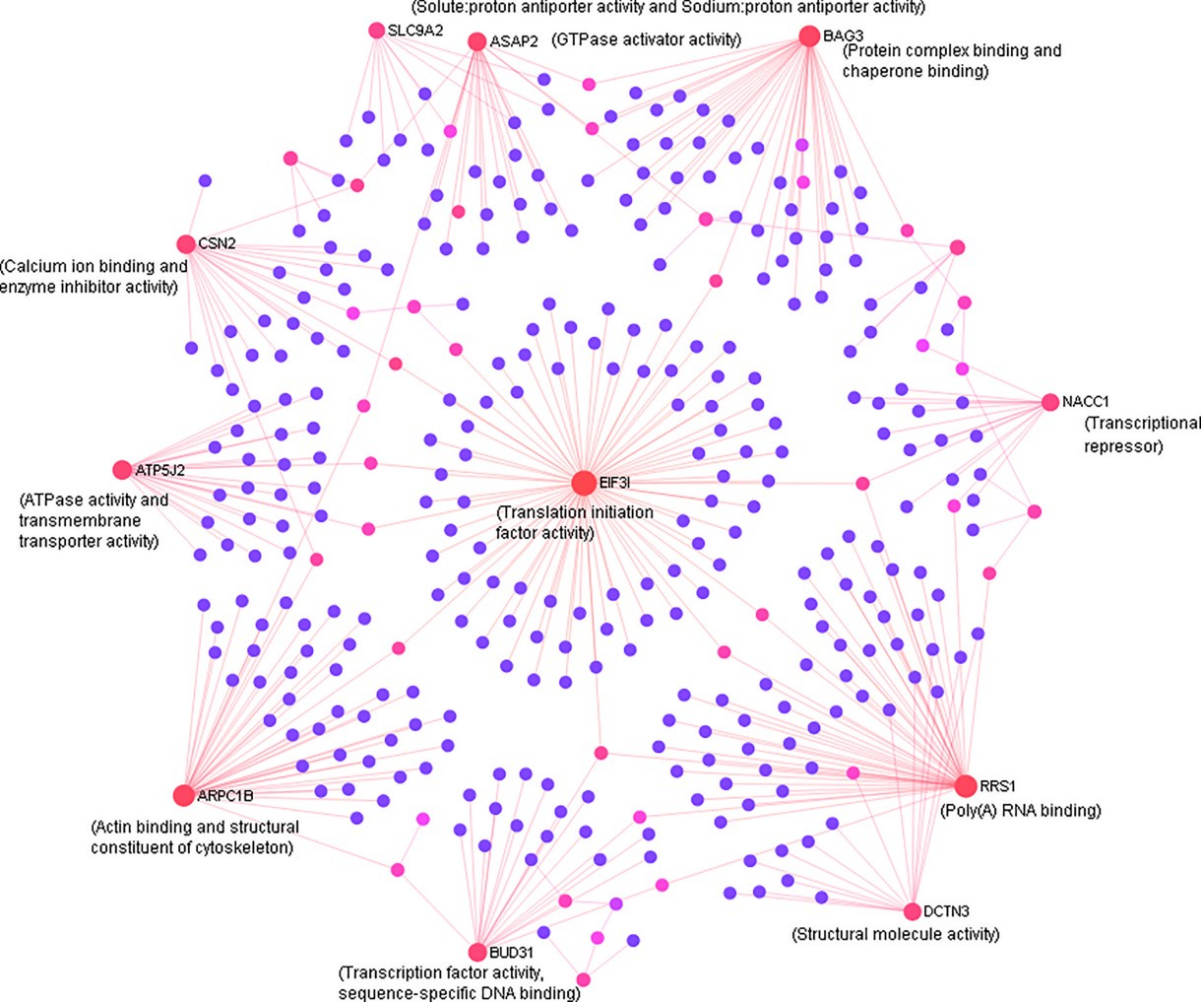
Network of commonly altered genes after PRRSV vaccination both in DL and Pi pigs. The interconnecting network showing the potential hub genes of the functional network of DEGs commonly observed in PBMCs of both DL and Pi pig at 24 h after PRRSV vaccination. Each circle of the network indicates node (seed gene) and the diameter of node accounted for its centrality estimates. Lines between nodes indicate the connectivity. The GO for corresponding genes are provided within the parenthesis. The network centrality estimates and relative expression values of major hub genes are provided in Table 1.

The transcripts showing differential expression shared between both breeds are summarized in S1 Table. To identify the potential regulatory genes among the common DEGs between two breeds, we performed the network enrichment analysis using the NetworkAnalyst tool [31]. The seed genes of the network were ranked based on their degree and betweenness centrality values to detect the most potential hub genes. The network of shared DEGs in PBMCs of both DL and Pi pigs is presented in Fig 4. Based on two centrality measures, the most highly interconnected hubs of functional network of the shared DEGs includes EIF3I (Eukaryotic translation initiation factor 3, subunit I), RRS1 (Ribosome biogenesis regulator homolog (S. cerevisiae)), ARPC1B (Actin related protein 2/3 complex, subunit 1B, 41kDa), BAG3 (BCL2-associated athanogene 3), ATP5J2 (ATP synthase, H+ transporting, mitochondrial Fo complex, subunit F2), CSN2 (Casein beta), ASAP2 (ArfGAP with SH3 domain, ankyrin repeat and PH domain 2), BUD31 (BUD31 homolog (S. cerevisiae)), DCTN3 (Dynactin 3 (p22)), NACC1 (Nucleus accumbens associated 1, BEN and BTB (POZ) domain containing) and SLC9A2 (Solute carrier family 9, subfamily A (NHE2, cation proton antiporter 2), member 2). Relative expression values and centrality estimates of the hub genes of shared transcriptome network are presented in Table 1. Surprisingly, the relative expressions of hub genes of shared network were opposite in direction between DL and Pi pigs. Among the hub genes, the relative expressions of SLC9A2, ASAP2, BAG3, NACC1, DCTN3, BUD31, ARPC1B and ATP5J2 were up-regulated in PBMCs of vaccinated DL pigs but down-regulated in PBMCs of vaccinated Pi pigs. In contrast, EIF3I was down-regulated in PBMCs of vaccinated DL pigs but up-regulated in those of Pi pigs. The expression of CSN2 was down-regulated in both breeds after vaccination, while RRS1 showed up-regulation in both breeds. Gene ontology (GO) annotations related to the hub genes include: protein complex binding, GTPase activity, sodium and hydrogen ion exchange, calcium ion binding, poly (A) RNA binding, structural molecule activity, transcription factor activity, actin binding, ATPase activity and translation initiation activity (Fig 4).

**Table 1:** Network centrality estimates and relative expression of major hub genes of transcriptome network shared between DL and Pi breeds.

Gene symbol	Network centrality		Fold changes	
	Degree	Betweenness	DL	Pi
EIF3I	79	25387	-1.743	1.771
RRS1	50	15702	2.005	1.619
ARPC1B	42	13702	1.769	-1.764
BAG3	39	12700	2.594	-1.664
ATP5J2	25	7719	2.468	-1.931
CSN2	22	9427	-3.041	-1.534
ASAP2	21	6625	2.435	-2470
BUD31	21	6495	2.202	-1.733
DCTN3	16	4970	1.780	-1.523
NACC1	16	4539	2.216	-1.607
SLC9A2	11	3279	2.340	-2.126

### Breed-specific transcriptome signatures for PRRSV vaccine responses

**Fig 5:**
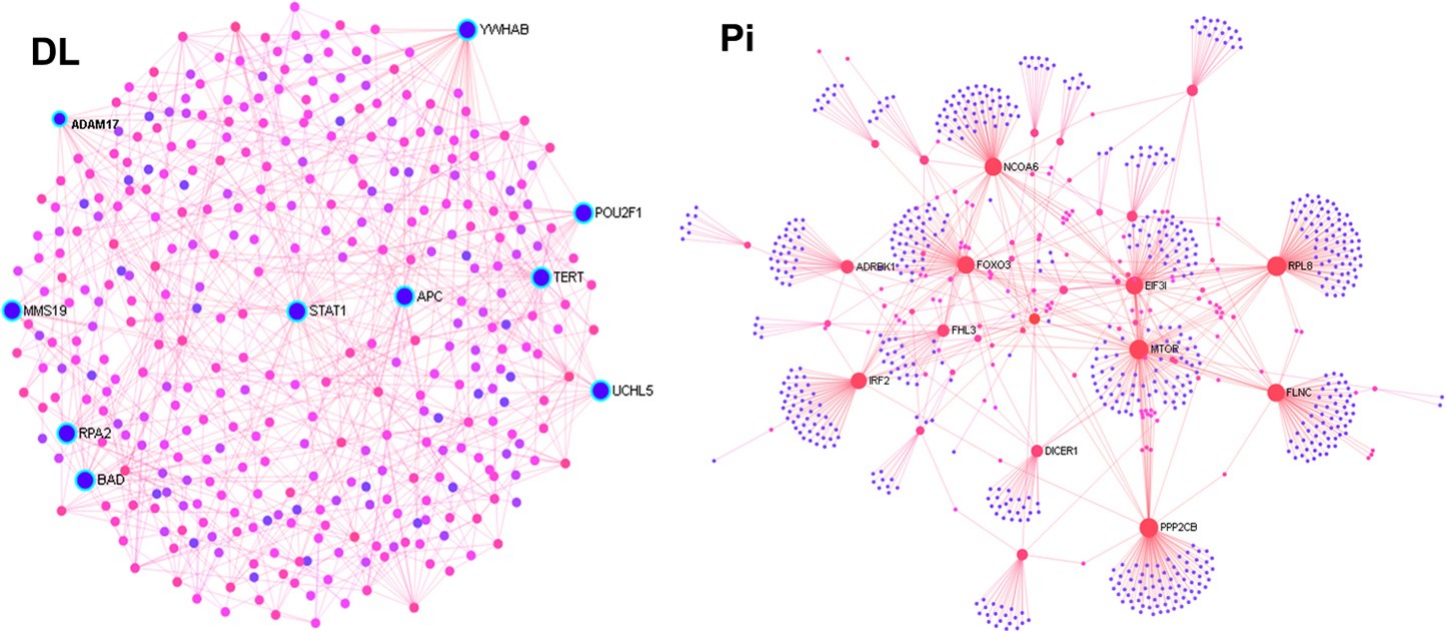
Network of breed-specific altered transcriptome in PBMCs after PRRSV vaccination. The network DL-specific DEGs is on the left panel (indicated by DL) and the Pi-specific transcriptome network is on the right panel (indicated by Pi). The network centrality estimates and relative expression values of major hub genes are provided in Table 2.

The breed-specific transcriptome signatures for PRRSV vaccine mediated immunity in PBMCs were identified through network enrichment analysis of vaccine-induced DEGs in DL and Pi pigs. The breed-specific transcriptome networks labeled with potential hub genes are presented in Fig 5. The degree and betweenness centrality estimates along with the relative expression of hub genes are provided in Table 2. The hub genes of DL specific transcriptome network include ADAM17 (Alpha disintegrin and metalloproteinase), STAT1 (Signal transducer and activator of transcription 1), MMS19 (MMS19 Homolog, cytosolic iron-sulfur assembly component), RPA2 (Replication protein A2), BAD (BCL2 associated agonist of cell death), UCHL5 (Ubiquitin C-terminal hydrolase L5) and APC (Adenomatous polyposis coli). While FOXO3 (Fork head box O3), IRF2 (Interferon regulatory factor 2), ADRBK1 (Adrenergic beta receptor kinase 1), FHL3 (Four and a half LIM domains 3), PPP2CB (Protein phosphatase 2 catalytic subunit beta), MTOR (Mechanistic target of rapamycin), EIF3I (Eukaryotic translation initiation factor 3 subunit), RPL8 (Ribosomal protein L8), DICER1 (Dicer 1, ribonuclease III), FLNC (Filamin C) and NCOA6 (Nuclear receptor coactivator 6) were found to be the most potential hubs of Pi specific transcriptome network.

**Table 2:** Relative expression and network centrality estimates of hub genes of breed-specific transcriptome networks.

Gene symbol	Fold changes	Network centrality	
		Degree	Betweenness
**DL-specific network**			
ADAM17	3.242	38	8043
STAT1	4.062	37	6073
MMS19	2.386	21	4344
BAD	2.604	19	3254
RPA2	2.645	18	3204
UCHL5	2.824	20	3163
APC	2.717	21	3122
**Pi-specific network**			
MTOR	3.214	7	282
FHL3	2.641	6	160
PPP2CB	2.712	6	97
EIF3I	3.812	5	90
RPL8	2.512	4	24
ADRBK1	2.971	4	16

### GO and pathways enriched by breed-specific DEGs

**Fig 6:**
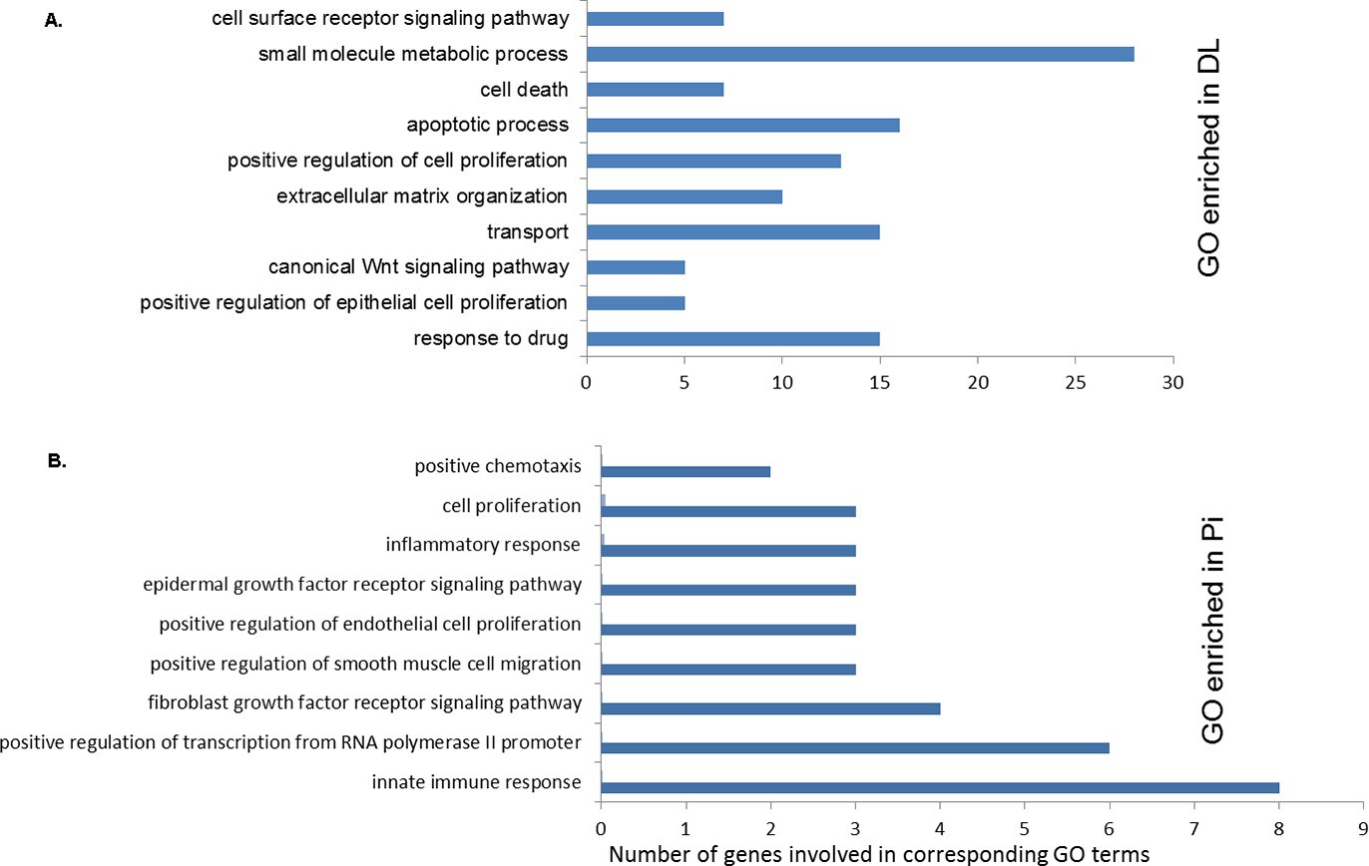
GO terms enriched by breed-specific DEGs. (A) Bar graphs showing the enriched GOs in the vaccinated PBMCs of DL compared to that of Pi (B) and GOs in the vaccinated PBMCs of Pi compared to that of DL. The p value of <0.05 was considered for statistically significant enrichment.

To elucidate the biological relevance of breed-specific host transcriptome alterations following PRRSV vaccination, we performed GO and pathway enrichment analyses for the genes showing unique differential expression in vaccinated PBMCs in two breeds using the InnateDB tool [29]. Among the breed dependent DEGs, 913 were more abundant in PBMCs of DL pigs (S2 Table), and 133 were more abundant in PBMCs of Pi pigs (S3 Table). Top GO terms enriched in vaccinated PBMCs of DL pigs compared to that of Pi pigs include cell surface receptor signaling pathways, small molecules metabolic process, apoptotic process, extracellular matrix organization, and response to drugs (Fig 6A, S4 Table). The GO for DEGs upregulated in vaccinated PBMCs of Pi pigs compared to that of DL pigs includes positive chemotaxis, cell proliferation, inflammatory responses, positive regulation of endothelial cell proliferation and innate immune response (Fig 6B, S5 Table).

Biological pathways significantly affected by genes differentially expressed in vaccinated PBMCs of DL pigs compared to those of Pi pigs include: signal transduction, protein metabolism, extracellular matrix organization, cytokine signaling in the immune system, interferon alpha/beta signaling and TNF receptor signaling pathway (Table 3, S6 Table). The pathways significantly altered by DEGs up-regulated in vaccinated PBMCs of Pi pigs compared to those of DL pigs include: innate immune system, signaling by FGFR in disease, TGF beta receptor, JAK-STAT pathway and regulation and chemokine signaling pathway (Table 3, S7 Table).

**Table 3:** Top ten biological pathways enriched by breed specific differentially expressed genes in PBMCs following PRRSV vaccination in DL and Pi pigs.

Pathway names	p-value	Genes involved
**DL-specific pathways**		
Signal Transduction	0.005	ADAM17, APC, APOE, B4GALT1, BAD, CNGA1, CRHR1,
		DNAL4, DRD2, DRD3, FLT4, FZD3, GFAP, GHRHR,
		GLP1R, GPR68, GREM2, LGR6,OR10H3, OR2AE1, OR4C46,
		OR4K13, OR4N2, OR6J1, OR7C2, OR9K2, PSME3, PTPRU,
		RDH8, RHOBTB2, SDC3, SDC4, SFRP1, SMO, STAT1,
		TERT, UCHL5, VIPR2 and YWHAB
Metabolism	0.05	ACSL6, ALDH2, APOE, ATP5J2, B4GALT1, CA12, CERS3,
		CYP17A1, DBT, DGUOK, DIO2, FBP1, GLP1R, GPAT2,
		HK3, IP6K1, KCNJ11, LRPPRC, LYPLA1, MED27, MMS19,
		MTMR7, NDUFS2, NDUFS3, NME2, PSME3, SDC3,
		SDC4 and SQLE
Extracellular matrix organization	0.008	ADAM17, BMP1, SDC3, SDC4, TGFB2, TLL1 and TLL2
Cytokine Signaling in Immune system	0.08	ADAM17, HLA-C, IFNA6, MX1, STAT1, TNIP2 and YWHAB
Wnt signaling pathway	0.002	APC, FZD3, SDC3, SDC4, SFRP1 and YWHAB
Apoptosis	0.007	ADAM17, APC, BAD, PSME3 and YWHAB
Glycolysis / Gluconeogenesis	0.02	ALDH2, ALDH3A1, FBP1 and HK3
Interferon alpha/beta signaling	0.05	HLA-C, IFNA60 and MX1
Antigen processing and presentation	0.08	HLA-C, HLA-DMB and PSME3
TNF receptor signaling pathway	0.09	ADAM17 and STAT1
**Pi-specific pathways**		
Innate Immune System	0.001	ADRBK1, FOXO3, IRF2, MTOR and PPP2CB
Signaling by FGFR in disease	0.001	ADRBK1, FOXO3,MTOR and PPP2CB
TGF beta receptor	0.02	EIF3I, FOXO3 and MTOR
JAK STAT pathway and regulation	0.03	ADRBK1, IL1A and MTOR
Chemokine signaling pathway	0.07	ADRBK1 and FOXO3
IL2 signaling events mediated by PI3K	0.003	FOXO3 and MTOR
Validated targets of C-MYC transcriptional-	0.01	DKK1 and FOXO3
repression		
Cell-Cell communication	0.004	CDH13, FLNC and KIRREL2
Glucose metabolism	0.01	GYS1 and PPP2CB
Platelet homeostasis	0.02	P2RX1 and PPP2CB

### Variation of PBMCs transcriptomes between unvaccinated DL and Pi pigs

Regardless of immunization, PBMCs transcriptome profiles of healthy control (before vaccination) pigs of DL and Pi breed also showed massive difference in transcript abundances (Fig 2). A complete list of DEGs in PBMCs of unvaccinated DL compared to that of unvaccinated Pi pigs is provided in S8 Table. The GO terms including ribosome, protein metabolism, catabolic process, cellular response to lipid, vasodilatation, phospholipid efflux and cartilage homeostasis were significantly enriched by the DEGs identified in PBMCs of unvaccinated DL pigs compared to those of Pi pigs. The DEGs were involved in enrichment of pathways including metal chelating activity, response to acetate, lactose biosynthetic process, tryptophan transport and visual behavior in the PBMCs of unvaccinated DL pigs compared to those of Pi pigs.

## Discussion

Innate host resistance to PRRS is becoming an area of great interest over the recent years because of the possibility for disease-resistant pig breeding. There is a consensus for genetic control of PRRS through improvement of host genetics by selective breeding for PRRS resistance [32]. However, data on innate host resistance to PRRSV, as measured by replication of the virus within the pig is very limited to date. To contribute in this scheme, one promising way to go is the identification of host genotypes associated with improved innate immune response following PRRSV vaccination [7]. In spite of considerably high heritability of disease resistance, only a little has been addressed in breeding programs, as these are difficult to measure [33]. Hence, an alternative approach of estimating disease resistance is recommendable through measuring host immunocompetence developed from vaccination [7]. To scrutinize the breed-specific transcripts for vaccine mediated immunity, we compared the global gene expression profiles of PBMCs collected before and after PRRSV vaccination in DL and Pi pigs.

Before vaccination, the healthy pigs of two breeds differed in their transcriptome profiles, which suggest that differential gene expression levels may be caused by the genetic differences between DL and Pi pigs, regardless of vaccine stimulation. Typically, DL pigs are more obese and heavier while Pi are lean-type pigs. Healthy DL and Pi pigs differ considerably in terms of growth rate, nutrient utilization and metabolic traits, as a result of their hepatic gene expression profiles [34]. Several researchers have also reported the genetic variation in immune traits between healthy pigs [33]. Differences in gene expression between the experimental groups, irrespective of infection, could be due to the different genetic makeup of two pig breeds. The variation in number as well as in function of neutrophils, monocytes and lymphocyte subsets in blood has been reported between healthy Meishan and Large White pigs [35]. Therefore, it is reasonable that the PBMCs transcriptome profiles of healthy DL and Pi pigs differ irrespective of immunization.

Following vaccination, the DL pigs used in this study significantly differed from Pi pigs in terms of transcriptional response to PRRSV vaccine, as evidenced by a higher number of DEGs in PBMCs of DL pigs than that of Pi pigs (Fig 2A). These differences on PRRSV vaccine induced global gene expression in PBMCs may be attributed by the host genotypes since the genetic configuration of each breed can display their specific pattern of coping strategy against stressors, which in turn leads to the variation of host resistance/susceptibility [34]. The global up-regulation of altered genes observed in vaccinated DL pigs (Fig 2B) was suggestive for PRRSV vaccine capacity to stimulate the immune system of DL pigs more effectively than Pi pigs. The current breed-specific patterns of gene expression changes in PBMCs at early post PRRSV vaccination are in agreement with the report of Ait-Ali et al. [15], who compared the microarray-based gene expression profiles of PRRSV infected lung tissue obtained from Landrace and Pi pigs. Landrace pigs showed a higher number of DEGs at 12 h post infection compared to that of Pi pigs [15]. In a similar fashion, differences on host susceptibility between DL and Pi pigs have also been reported in response to porcine circovirus infection [36]. The gene expression differences observed here may be due to the genotype variation, demonstrated how the different breeds react to the vaccine exposure within the 24 h of administration. However, antibody responses at 4 weeks post vaccination were not significantly differed between DL and Pi pigs [24, 25]. The gene expression differences observed here may be due to the genotype variation, demonstrating how the different breeds react to the vaccine exposure within the 24 h. Perhaps for the generalized nature of innate immunity, these early stage gene expression changes may not necessarily be similar with the vaccine-specific antibody response in the later stages. Therefore, it is important to further elucidate the correlation between the DEGs phenotype early (hours) after vaccination and other phenotypes such as the vaccine-specific antibody response, response to infection or ability to protect from challenge, at later stages (2–6 weeks) of vaccination in the same individual. Even though it is not clear yet whether host responses based on measurable pathogenesis is better than those based on protective attributes, transcriptome signature of innate immune response to vaccination is of great importance because genes regulating the early host response to vaccination, as mimicking the immune response to pathogenic infection [21], are likely to be the potential candidate for host’s resistance to disease [5].

PRRSV vaccination resulted in a global down-regulation of transcriptomes in PBMCs of Pi pigs (Fig 2B), indicating the suppression of immune system functions at 24 h post vaccination. This is comparable to one of our previous studies, where we observed a global down-regulation of altered transcriptomes in PRRSV infected dendritic cells obtained from Pi pigs compared to those of Duroc pigs [19]. We observed that ubiquitin associated protein 2 and ubiquitin specific protease 45 were up-regulated in the PBMCs of DL pigs while the member of ubiquitin family USP18 is known to be associated with host resistance to PRRS [12]. Variation of susceptibility to PRRSV infection has also been reported in several comparisons among the pigs obtained from purebreds and crossbreds [14, 18]. Hampshire-Duroc crossbred pigs were found to be more susceptible to in vivo PRRSV infection than pigs of NE Index lines [17]. Similarly, purebred Hampshire pigs showed significantly more severe lung lesions after in vivo PRRSV infection than Duroc or Meishan pigs [14]. Therefore, variations observed between PBMCs transcriptome profiles of DL and Pi pigs after PRRSV vaccination was a strong indication for breed differences in host response to PRRSV. However, it should be considered that the present in vivo study was based on a single time point (24 h post vaccination) and three biological replications in each breed. Though an in vitro study has proposed 24 h post stimulation as a reference time point for exploring the transcriptional modifications in PBMCs linked to innate immune responses [37], a time series investigation including higher number of animals and with more immunologically focused transcriptome analyses between DL and Pi pigs would be rationale to confirm the genetic differences in vaccine-induced transcriptome modifications.

Like other quantitative traits, immune response traits are likely regulated by multiple genes which interact with each other through an interconnecting network [38]. Therefore, network-based approaches have been considered more sensitive to find the regulatory gene molecules for global transcriptome alterations [39]. Herein we performed the network analysis to scrutinize the regulatory genes from the list of vaccine induced DEGs which were common in PBMCs of both breeds (Fig 4), DEGs which were more abundant in DL (Fig 5, left) and DEGs which were more abundant in Pi pigs (Fig 5, right). The predicted hub genes of a transcriptome network are likely to promote or inhibit the expression of other connecting genes to maintain the biological function [39]. Many genes showed differential expression in PBMCs of both DL and Pi pigs after PRRSV vaccination, but their directions (up or down) of expression changes were opposite in two breeds. Eight out of top ten hub genes of the shared network (Fig 4) were up-regulated in DL, while only EIF3I was up-regulated in Pi, only CSN2 was down-regulated in both breeds and RRS1 was mutually up-regulated in both breeds (Table 1). This was an indication of functional variation even within the common DEGs between breeds. Opposition in expression regulation of the same genes between breeds, may be caused by the variation of functional regulation of individual genes as reported by Xing et al. [13]. The GO terms linked to hub genes of the shared network indicated their involvement in cellular immune response to infection (Fig 4). The genomic locations of hub genes are distributed over SSC 1, 3, 4, 7 and 5, while the QTLs for PRRSV susceptibility have already been identified on SSC 1, 4 and 7 [9, 40]. Among the hub genes, SLC9A2, NACC1 and EIF3I are known to be involved in cancerous growth [41] while RRS1, DCTN3, ARPC1B and BAG3 are reported to be associated with host immune response [42, 43]. However, to our knowledge, these genes have not yet been functionally linked well to the PRRSV vaccine mediated immunity in pig. It is therefore important to analyze the expression patterns of these hub genes in other porcine breeds following PRRSV vaccination.

Involvement of DL-specific network hub genes (Fig 5) in the enrichment of pathways including signal transduction, extracellular matrix organization, cytokine signaling, apoptosis and TNF receptor signaling pathway (Table 3), indicated their potentiality to regulate the PRRSV vaccine-induced innate immunity. Among the hub genes of DL-specific network, ADAM17 was significantly up-regulated in PBMCs of DL pigs (Table 2) and participated in most of the DL-specific enriched pathways (Table 3). ADAM17 is one of the best characterized of the ADAM enzymes, functionally involved in ectodomain shedding, a post-translational modification, of various transmembrane proteins: EGFR ligands, proinflammatory cytokines like TNF and its receptor TNFRI, adhesion molecules and the amyloid precursor protein like APP (reviewed in [44]). ADAM17 takes part in the proinflammatory process through ectodomain shedding of the TNF precursor [45] and cell proliferation through activating EGFR [46]. Especially, ADAM17 mediates the down-regulation of CD163 expression [47], a putative cellular receptor that facilitates the entrance of PRRSV into host macrophages through endocytosis [48]. Therefore, the overexpression of ADAM17 in DL pigs compared to Pi pigs could explain the relatively higher PRRS-resistance capacity of DL pigs. Next to ADAM17, another DL-specific hub gene, STAT1, a protein coding gene of the signal transducer and transcription activator (STAT) protein family, was more abundant in PBMCs of DL pigs compared to those Pi pigs after PRRSV vaccination. The STATs mediate the cellular responses to interferons (IFNs), cytokines and other growth factors involved in antiviral innate immunity [49], and have been linked with PRRSV immunity [50]. Another hub gene MMS19 is an essential component of the cytoplasmic iron-sulfur (Fe-S) cluster assembly complex in fungi and mammals, having functions in nucleotide excision repair, a major cellular defense mechanism against DNA damage [51], indicating its potential to be involved with PRRSV immunity. Likewise, functional involvement of other hub genes including RPA2 [52] and BAD [53] in cellular immunity suggested their potential role in PRRSV vaccine induced transcriptome alterations in DL pigs.

On the other hand, FOXO3 was found to be a highly interconnected hub gene in the PBMCs of PRRSV vaccinated Pi pigs as compared to those of DL pigs. FOXO3 belongs to the fork head family of transcription factors and functions as a trigger for apoptosis through expression of genes necessary for cell death and participates in post-transcriptional regulation of MYC transcription factor [54] which has been reported to be associated with host response to PRRSV [55]. Next to FOXO3, IRF2 was also up-regulated in PBMCs of Pi pigs which is known to be associated with suppression of Type I interferon response, a potent antiviral innate immunity [56]. Among other Pi-specific hub genes: ADRBK1 is known to be involved in neuroinflammation and multiple sclerosis [57]; FHL3 is involved in regulating integrin-mediated cytoskeletal events [58]; PPP2CB is known to be involved in bladder cancer [59]. Although, most of the hub genes of both DL- and Pi-specific transcriptional networks were individually involved with the immune response trait, however, they exert function through participating in different pathways and in different directions also (Table 3). These suggested that the immune system of DL pigs responded to gene expression changes in such a way that differed from Pi pigs.

Activation of PRRSV vaccine induced interferon response was more prominent, at least to some extent, in the DL pigs compared to those of Pi pigs as indicated by up-regulation of STAT1 as in DL-network, and by down-regulation of IRF2 in the Pi-network. Moreover, the interferon alpha/beta pathways (IFN6, MX1) were up-regulated in PBMCs of vaccinated DL pigs (Table 3) but not in the case of Pi pigs. The type I IFNs secreted from vaccine-pulsed PBMCs, may interact with a subset of naive T-cells to promote their conversion into virus-specific IFN secreting cells, thereby inducing the cell mediated interferon response [60]. The induction of a type I interferon response was reported to be observed early after in vitro PRRSV infection in alveolar macrophages of Landrace pigs [15]. Concordantly, the expression of myxovirus resistance 1 (MX1) has been reported to be up-regulated in PRRSV infected pulmonary alveolar macrophages over 24 h post infection period [61]. We therefore speculate that the potential network hubs identified might play an important role in the host susceptibility/resistance to PRRSV infection.

## Conclusions

This study reported the host genetic variation in PRRSV vaccine mediated innate immunity between DL and Pi pigs using genome-wide transcriptome profiles of PBMCs. A higher number of gene transcripts showed differential expression in DL pigs early after PRRSV vaccination as compared to those of Pi pigs. Functional analyses of breed-specific alterations elucidated the differences in molecular mechanisms of PRRSV vaccine-induced innate immunity development between DL and Pi pigs. Notably, over expression of ADAM17 in PBMCs of vaccinated DL pigs is an indication for relatively higher PRRS-resistance capacity as compared to that of Pi pigs. ADAM17 plays crucial role in blocking the PRRSV entrance to macrophage through down-regulating the CD163 expression. Although, our data provide an evidence of breed-specific immunity and valuable information for improving PRRS resistance in pigs, time series investigation including higher number of animals and with more immunologically focused transcriptome analyses between DL and Pi pigs could be carried out to get further insights on genetic contribution of the anti-PRRSV immunity in pigs. In addition, exploring the correlation between immunotranscriptome alterations in the early (hours) stage and other immune response phenotypes such as vaccine-specific antibody titre, response to infection or ability to protect from challenges in the later stages (2–6 weeks) of vaccination in the same individual are also interesting topics for future research.

## Supporting information

S1 Table List of DEGs expressed in PBMCs of both DL and Pi pigs at 24 h post PRRSV vaccination compared to their respective controls. (XLSX)

S2 Table DEGs more abundant in vaccinated PBMCs of DL pigs compared to that of Pi pigs. (XLSX)

S3 Table DEGs more abundant in vaccinated PBMCs of Pi pigs compared to that of DL pigs. (XLSX)

S4 Table GO terms enriched by DEGs more abundant in vaccinated PBMCs of DL pigs. (XLSX)

S5 Table GO terms enriched by DEGs more abundant in vaccinated PBMCs of Pi pigs. (XLSX)

S6 Table Pathways enriched by DEGs more abundant in vaccinated PBMCs of DL pigs. (XLSX)

S7 Table Pathways enriched by DEGs more abundant in vaccinated PBMCs of Pi pigs. (XLSX)

S8 Table List of DEGs in unvaccinated PBMCs of healthy DL pigs compared to that of Pi pigs. (XLSX)
